# Survival analysis of diagnostic assays in *Plasmodium falciparum* malaria

**DOI:** 10.1186/s12936-015-0882-1

**Published:** 2015-09-17

**Authors:** Melissa Phuong, Rachel Lau, Filip Ralevski, Andrea K. Boggild

**Affiliations:** Faculty of Health Sciences, McMaster University, Hamilton, Canada; Public Health Ontario Laboratories, Public Health Ontario, Toronto, Canada; Tropical Disease Unit, Division of Infectious Diseases, UHN-Toronto General Hospital, 200 Elizabeth Street, 13EN-218, Toronto, ON M5G 2C4 Canada; Department of Medicine, University of Toronto, Toronto, Canada

**Keywords:** *Plasmodium falciparum*, Malaria diagnosis, PCR, Rapid antigen detection test

## Abstract

**Background:**

Rapid diagnostic tests (RDT) and real-time PCR (qPCR) assays are sensitive for diagnosing malaria, but because they detect antigen and DNA, respectively, positivity may not reflect active infection. Performance characteristics of RDT and qPCR in *Plasmodium falciparum* positive specimens were evaluated over time to elucidate duration of positivity following conversion to microscopy negative.

**Methods:**

Specimens from patients with at least one specimen that was positive for *P. falciparum* by microscopy, and at least one specimen that was negative for *P. falciparum* within a 1-month period were identified. Survival distributions of the diagnostic tests over time were compared. Performance characteristics for each test were calculated.

**Results:**

Ninety specimens were included, with 48 initially positive for *P. falciparum*, and 42 subsequently negative. Of 42 specimens that converted to microscopy-negative following an initial positive, 26 (61.9 %) and 41 (97.6 %) were positive by qPCR and RDT, respectively. Survival curves of microscopy versus qPCR, as well as microscopy vs RDT differed significantly (p = 0.0002 and p < 0.0001, respectively). Compared to microscopy, sensitivity of qPCR was 100.0 % (95 % CI 90.8–100.0 %), and that of RDT was 100.0 % (95 % CI 90.8–100.0 %).

**Conclusions:**

Due to slow clearance of circulating antigen and DNA from bloodstream, RDT and qPCR have low positive predictive value for clinically relevant asexual parasitaemia in post-treatment specimens. Thus, microscopy remains the only available malaria diagnostic that can reliably distinguish true asexual parasitaemia from prolonged clearance of antigen and nucleic acid in a convalescing patient.

## Background

Three common methods are used to diagnose malaria: microscopic examination of Giemsa-stained thick and thin blood smears; rapid diagnostic tests (RDTs); and polymerase chain reaction (PCR) or other nucleic-acid based assays [1, 2]. These tests have varying degrees of sensitivity and specificity for different *Plasmodium* species [2, 3], however, they are highly sensitive for detecting *Plasmodium falciparum*, as species-specific morphology, histidine-rich protein 2 (HRP-2), and species-specific 18S rRNA gene can all be detected or amplified [1, 4].

Microscopy remains the gold standard diagnostic test for malaria due to its sensitivity and specificity in expert microscopist hands, although considerable expertise is required, and results are operator dependent [2, 4]. RDTs are routinely implemented due to their relative simplicity and rapid turnaround time, enabling them to function as a point-of-care diagnostic. The specificity of RDTs can be low, particularly in the setting of persistent antigenaemia post-treatment of *P. falciparum* infection, as well as non-*falciparum* species [2, 5–7]. More recently, real-time polymerase chain reaction (qPCR) has become increasingly implemented, although its availability is limited to well-resourced reference facilities. qPCR assays have high sensitivity and specificity, and they may be used to confirm ambiguous microscopic results as well as identify the presence of mixed infections [1]. Given that RDTs and qPCR detect parasite antigen and DNA, respectively, specificity can be compromised if clearance of these parasite components from the bloodstream of the patient is slow [5, 7].

The aim of this study was to examine the length of time that qPCR and RDT results remain positive in specimens from patients recovering from recent *P. falciparum* infection. The natural history of HRP-2 and 18S DNA clearance relative to microscopy is herein described in a series of malaria-positive specimens submitted to our laboratory.

## Methods

### Specimens

Surplus and anonymized malaria-positive specimens entered into the malaria biobank were identified and included in the analysis if: multiple specimens from a single individual were entered with not more than 1-month between the first and second specimen; isolated *P. falciparum* malaria was confirmed microscopically for the first specimen, with conversion to microscopic negativity or declining parasitaemia on at least one subsequent specimen; complete RDT (BinaxNOW Malaria kit, Alere, ME) and Giemsa-stained thick and thin film microscopy results within the biobank database; and submission to the laboratory between October 2008 and April 2014.

### DNA extraction

DNA was extracted from banked whole blood specimens using DNA Mini Kit Blood or Body Fluid Spin Protocol (Qiagen, Germantown, MD, USA). For each specimen, 200 μL of frozen whole blood was thawed from −80 °C and DNA was eluted with 60 μL AE buffer. DNA was then stored at −20 °C prior to amplification.

### Qualitative real-time PCR

All specimens underwent the following qPCR assays: human beta-2-microglobulin (B2MG) extraction control, *Plasmodium malariae*/*Plasmodium ovale* species-specific duplex and *P. falciparum*/*Plasmodium vivax* species-specific duplex, as previously described [8]. *Plasmodium falciparum*/*P. vivax* species-specific duplex qPCR assay was conducted to confirm the isolated presence of *P. falciparum* and quantify copy number of the 18S rRNA gene. *Plasmodium malariae*/*P. ovale* species-specific duplex qPCR was conducted to exclude initial mixed infection as well. All qPCR assays were run using ABI 7900HT Real-time PCR System using the following standard conditions: 50 °C for 2 min, 95 °C for 10 min, 95 °C for 15 s, and 60 °C for 1 min (45 cycles), as previously described [8]. For each 25-µL reaction, 12.5 µL of TaqMan universal PCR master mix (Life Technologies), 7.5 µL of primer and probe mixes with concentrations previously reported [1, 2, 8] and 5 µL of DNA were used.

All qPCR amplification curves were analysed using a manual Ct threshold of 0.02 and an automatic baseline. Positive results were identified if the Ct value was <40 for the B2MG and *P. falciparum*/*P. vivax* species-specific duplex assays or <38 for the *P. malariae*/*P. ovale* species-specific duplex assay, as previously described [8].

### Quantitative real-time PCR

In order to quantify gene copy number, serial dilutions of a *P. falciparum* 18S rRNA DNA clone (MRA-177 ATCC^®^ 83, Virginia) that ranged from 9 to 91 million copies/qPCR reaction were prepared and were included in each run of the *P. falciparum*/*P. vivax* species-specific duplex qPCR assay. The logarithm of gene copy number was plotted against Ct values for each concentration of the clone. A linear regression was conducted from this graph, and the equation was used to calculate gene copy number for each banked specimen.

### Statistical analysis

The number of days between each specimen in a group of specimens for a single case were calculated. Kaplan–Meier survival curves were constructed, where an event was defined as either negative microscopy, qPCR, or RDT result. The alpha level (α) was predetermined to be 0.05 for the log-rank test of all three diagnostic procedures, and a Bonferroni correction was applied for post hoc analysis among the three diagnostic tests.

To evaluate test performance characteristics for subsequent samples received after the initial specimen, two sets of calculations were conducted: one using microscopy as the reference standard, and one using qPCR as the reference standard. For each scenario, sensitivity, specificity, positive predictive value (PPV), and negative predictive value (NPV) were calculated. Analysis was conducted using IBM SPSS Statistics (IBM, New York, USA) and graphs were prepared using GraphPad Prism 5 (GraphPad Software, La Jolla, CA, USA).

## Results

In total, 90 specimens from 24 individuals entered into the malaria biobank were analysed.

### Microscopy, rapid diagnostic test, and real-time PCR comparison

Twenty-four initial specimens and 66 subsequent specimens were received from 24 patients, where 48 initial specimens were positive for *P. falciparum* by microscopy, with 42 subsequently collected specimens converting to microscopy negative. Out of 90 specimens, 89 (98.9 %) were positive for HRP-2 by RDT, thus, 41 microscopy-negative specimens (97.6 %) were positive by RDT. Seventy-four specimens (82.2 %) were positive by qPCR, thus 26 microscopy negative specimens (61.9 %) were positive by qPCR.

**Fig. 1:**
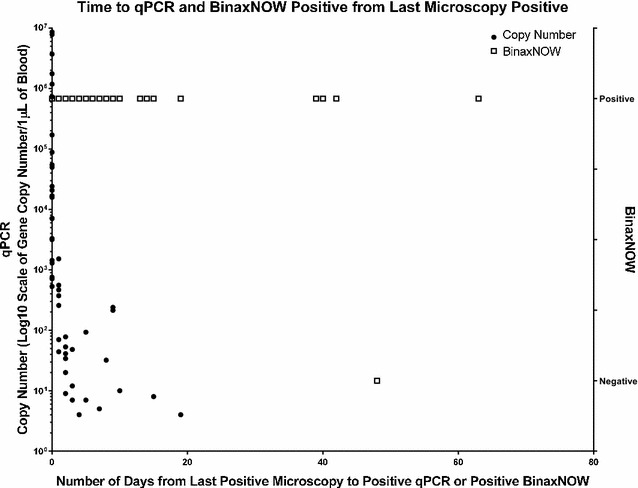
Number of days between the time from last *P. falciparum*-positive specimen by microscopy was collected and the time when subsequent specimens which were microscopically negative but positive by either qPCR or BinaxNOW were collected for each case. Copy number from each specimen in each reaction was plotted for qPCR. For RDT, 1.0 indicates a positive result, whereas −1.0 indicates a negative result

**Fig. 2:**
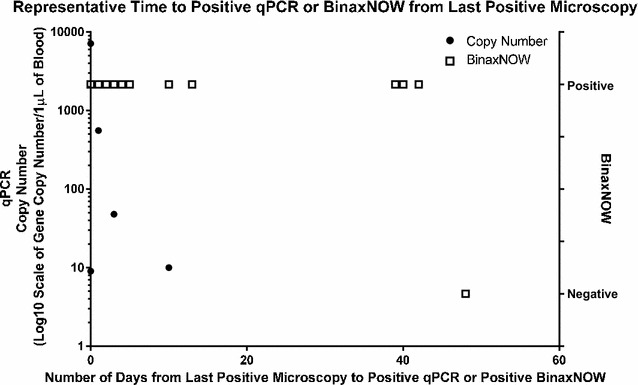
Positivity of qPCR and RDT results following initial confirmation of *P. falciparum*-negative conversion by microscopy in two sets of specimens with frequent samples submitted over a 1-month period

Time between the last *P. falciparum*-positive microscopic result, positive qPCR, and RDT is summarized in Fig. 1. qPCR and RDT remained positive up to 19 and 63 days following microscopic conversion to negativity, respectively. In two cases, multiple specimens submitted over a period of 2 months, and time between the last *P. falciparum*-positive microscopic result, positive qPCR, and RDT for these two cases with multiple specimens is summarized in Fig. 2.

**Fig. 3:**
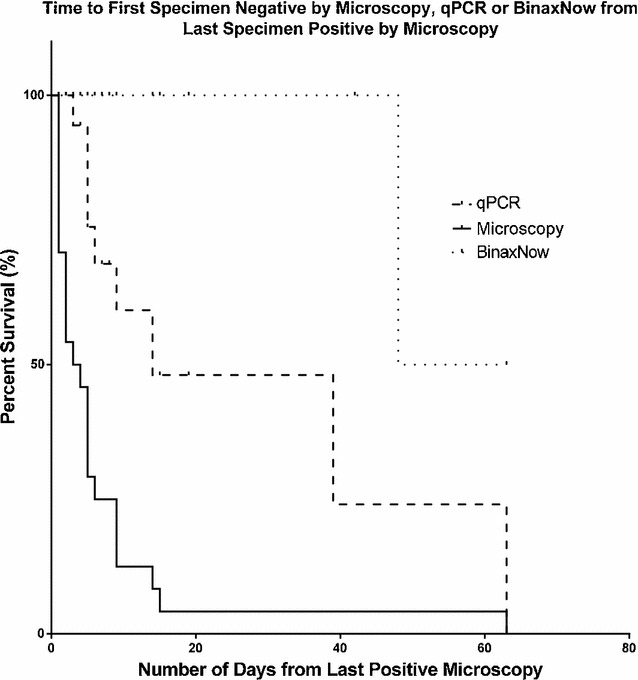
Kaplan–Meier survival curves for all three diagnostic assays

The three survival distributions (Fig. 3) were found to be significantly different by log-rank test [χ^2^(2) = 39.62, p < 0.0001]. Specifically, survival distributions of microscopy and qPCR, as well as microscopy and RDT were found to differ significantly [χ^2^(1) = 14.11, p = 0.0002 and χ^2^(1) = 31.79, p < 0.0001, respectively].

Performance characteristics of qPCR and RDT compared to microscopy for samples received after the initial specimen are summarized in Table 1. Sensitivity and specificity of qPCR were 100.0 % (95 % CI 82.8–100.0 %), and 38.1 % (95 % CI 24.0–54.3 %), respectively. PPV for qPCR was 48.0 % (95 % CI 33.9–62.4 %) and NPV was 100.0 % (95 % CI 75.9–100.0 %). Compared to microscopy, sensitivity of RDT for asexual parasitaemia was 100.0 % (95 % CI 82.8–100.0 %) and specificity was 2.4 % (95 % CI 0.1–14.1 %). PPV and NPV for RDT were 36.9 % (95 % CI 25.6–49.8 %) and 100.0 % (95 % CI 5.5–100.0 %) compared to microscopy, respectively.

**Table 1:** Performance characteristics of qPCR and BinaxNOW with microscopy as the reference standard for subsequent specimens

	Reference standard: microscopy			
	True positives	True negatives	False positives	False negatives
qPCR	24	16	26	0
BinaxNOW	24	1	41	0

Performance characteristics of microscopy and RDT compared to qPCR are summarized in Table 2. Sensitivity and specificity of microscopy were 75.8 % (95 % CI 63.4–85.1 %) and 100.0 % (95 % CI 75.9–100.0 %), compared to qPCR, respectively. PPV was 100.0 % (95 % CI 82.8–100.0 %) and NPV was 38.1 % (95 % CI 24.0–54.3 %). Compared to qPCR, sensitivity of RDT was 100.0 % (95 % CI 91.1–100.0 %) and specificity was 6.3 % (95 % CI 0.3–32.3 %). PPV and NPV for RDT were 98.5 % (95 % CI 64.5–86.1 %) and 100.0 % (95 % CI 5.5–100.0 %) compared to qPCR, respectively. RDT had a very low specificity and PPV for asexual *P. falciparum* parasitaemia, regardless of comparator.

**Table 2:** Performance characteristics of microscopy and BinaxNOW with qPCR as the reference standard for subsequent specimens

	Reference standard: qPCR			
	True positives	True negatives	False positives	False negatives
Microscopy	24	16	0	26
BinaxNOW	50	1	15	0

## Discussion

Of three main diagnostic platforms—microscopy, PCR, and RDT—microscopy is considered the gold standard [9, 10]. Furthermore, microscopy is the only available diagnostic test for malaria that can reliably differentiate sexual from asexual parasitaemia. As PCR and RDT detect parasite components rather than the whole parasite itself, clearance from the bloodstream may be delayed [2, 7], and may yield false-positive results post-treatment. In addition, sexual stages of *P. falciparum* (gametocytes), which have no clinical relevance, may lead to positive RDT and/or qPCR, and this may be misinterpreted by clinicians as relapse or recrudescence of asexual parasitaemia. Recommendations to perform day-7 and day-28 follow-up parasitaemia levels in falciparum malaria [11], were made prior to the widespread use of RDTs and qPCR assays in hematology and reference laboratories, and must be interpreted accordingly. Understanding the performance limitations of RDT and qPCR is imperative to correct interpretation of malaria diagnostic tests in clinical cases of malaria, particularly following appropriate administration of therapeutic anti-malarials. As laboratories are often not privy to ongoing clinical details in falciparum malaria, careful reporting of RDT and qPCR results must be undertaken, with sufficient exposition of limitations, particularly in the context of microscopic conversion to negativity.

Through survival analysis, we have documented the significant time difference in clearance of whole parasites, DNA, and HRP-2 following initial reporting of *P. falciparum* asexual parasitaemia. This study is limited in its ability to provide a robust estimate of clearance time for each test due to the small number of enrolled specimens and collection at different time points following the initial positive test. Furthermore, there is the potential for bias in the studied sample, as uncomplicated cases or patients who are semi-immune may be less likely to return for follow-up testing. Thus, more rapid parasite DNA or HRP-2 clearance may by under-represented in this study. Future studies with prospective enrolment of patients and daily follow-up of microscopic parasitaemia, RDT, and qPCR would address this limitation and provide a more precise estimate of clearance for each test.

## Conclusions

Microscopy, qPCR, and RDT have variable performance characteristics and taken together, provide highly sensitive and specific detection of *P. falciparum* asexual parasitaemia. For follow-up post-treatment, however, microscopy still remains the only assay with adequate specificity for ongoing asexual parasitaemia and with low relative risk of false positivity. Laboratories must be cognizant of the RDT and qPCR performance limitations that, if reported in isolation, may lead to misinterpretation of results and potential erroneous re-treatment of malaria in a clinically-well patient free of asexual parasitaemia.
